# An Integrated Molecular Approach to Untangling Host–Vector–Pathogen Interactions in Mosquitoes (Diptera: Culicidae) From Sylvan Communities in Mexico

**DOI:** 10.3389/fvets.2020.564791

**Published:** 2021-03-10

**Authors:** Luis M. Hernández-Triana, Javier A. Garza-Hernández, Aldo I. Ortega Morales, Sean W. J. Prosser, Paul D. N. Hebert, Nadya I. Nikolova, Elsa Barrero, Erick de J. de Luna-Santillana, Vicente H. González-Alvarez, Ramón Mendez-López, Rahuel J. Chan-Chable, Anthony R. Fooks, Mario A. Rodríguez-Pérez

**Affiliations:** ^1^Animal and Plant Health Agency, Virology Department, Rabies and Wildlife Zoonoses Research Group, Addlestone, United Kingdom; ^2^Instituto de Ciencias Biomédicas, Universidad Autónoma de Ciudad Juárez, Chihuahua, Mexico; ^3^Departamento de Parasitología, Universidad Autónoma Agraria Antonio Narro, Unidad Laguna, Periférico Raúl López Sánchez y Carretera a Santa Fe, Torreón, Mexico; ^4^Center for Biodiversity Genomics, University of Guelph, Guelph, ON, Canada; ^5^Instituto Politécnico Nacional, Centro de Biotecnología Genómica, Reynosa, Mexico; ^6^Facultad de Medicina Veterinaria y Zootecnia, Universidad Autónoma de Guerrero, Chilpancingo, Mexico

**Keywords:** bloodmeals, mosquitoes, cytochrome c oxidase I, DNA barcoding, chiapas state, Mexico

## Abstract

There are ~240 species of Culicidae in Mexico, of which some are vectors of arthropod-borne viruses such as Zika virus, dengue virus, chikungunya virus, and West Nile virus. Thus, the identification of mosquito feeding preferences is paramount to understanding of vector–host–pathogen interactions that, in turn, can aid the control of disease outbreaks. Typically, DNA and RNA are extracted separately for animal (insects and blood meal hosts) and viral identification, but this study demonstrates that multiple organisms can be analyzed from a single RNA extract. For the first time, residual DNA present in standard RNA extracts was analyzed by DNA barcoding in concert with Sanger and next-generation sequencing (NGS) to identify both the mosquito species and the source of their meals in blood-fed females caught in seven sylvan communities in Chiapas State, Mexico. While mosquito molecular identification involved standard barcoding methods, the sensitivity of blood meal identification was maximized by employing short primers with NGS. In total, we collected 1,634 specimens belonging to 14 genera, 25 subgenera, and 61 morphospecies of mosquitoes. Of these, four species were new records for Mexico (*Aedes guatemala, Ae. insolitus, Limatus asulleptus, Trichoprosopon pallidiventer*), and nine were new records for Chiapas State. DNA barcode sequences for >300 bp of the COI gene were obtained from 291 specimens, whereas 130 bp sequences were recovered from another 179 specimens. High intraspecific divergence values (>2%) suggesting cryptic species complexes were observed in nine taxa: *Anopheles eiseni* (5.39%), *An. pseudopunctipennis* (2.79%), *Ae. podographicus* (4.05%), *Culex eastor* (4.88%), *Cx. erraticus* (2.28%), *Toxorhynchites haemorrhoidalis* (4.30%), *Tr. pallidiventer* (4.95%), *Wyeomyia adelpha/Wy. guatemala* (7.30%), and *Wy. pseudopecten* (4.04%). The study increased the number of mosquito species known from 128 species to 138 species for Chiapas State, and 239 for Mexico as a whole. Blood meal analysis showed that *Aedes angustivittatus* fed on ducks and chicken, whereas *Psorophora albipes* fed on humans. *Culex quinquefasciatus* fed on diverse hosts including chicken, human, turkey, and Mexican grackle. No arbovirus RNA was detected by reverse transcriptase–polymerase chain reaction in the surveyed specimens. This study demonstrated, for the first time, that residual DNA present in RNA blood meal extracts can be used to identify host vectors, highlighting the important role of molecular approaches in both vector identification and revealing host–vector–pathogen interactions.

## Introduction

The family Culicidae is medically important because of the large number of pathogens that some species transmit to animals and humans, and it is also a driver of numerous emerging infectious diseases around the world (1, 2). Knowledge of the blood-feeding preferences of a mosquito species provides important insight into the dynamics of virus transmission, allowing public health authorities to design and implement efficient strategies for vector control (3). Mosquito-vectored pathogens contribute to the greatest diversity of neglected tropical diseases that significantly impact human and animal health (4). There are 3,574 recognized species of Culicidae worldwide (5), so correct identification of the species that act as vectors is critical for characterizing pathogen transmission pathways.

Host selection and feeding preference studies of mosquitoes and other hematophagous arthropods, in combination with pathogen screening play a major role in understanding the dynamics of vector–host–pathogen interactions (6–16). Once the feeding preferences are known, and host species at risk of transmitting arthropod-borne pathogens are identified, the mechanisms of disease transmission can be elucidated (17–19). Systematic characterization of bird and mammalian host genetics has increased the specificity of studies. Driven by the use of molecular techniques, genetic analysis has largely replaced serological methods for blood meal identification (9). Several genetic markers have been used for this purpose, including mitochondrial (e.g., cytB, COI) and nuclear (e.g., ITS2) (20, 21) markers.

While genetic analysis has largely replaced serological methods, host-preference studies face challenges. First, the accurate identification of arthropod vectors is complicated by the morphological similarity of species, by decreasing taxonomic expertise, and by the presence of species complexes (22–25). Second, the capacity to recover a sequence for the host is affected by the degree of digestion of the blood meal within the mosquito, as well as the method of preservation after capture (15, 16, 26). Third, the potential presence of pathogens within the blood meal increases biosafety issues. To overcome the first barrier, analysis of the COI mtDNA barcode region (27, 28) is now widely used for mosquito identifications worldwide (29–34). To mitigate the second challenge, researchers now employ high-throughput sequencing in combination with vertebrate-specific primer cocktails (35, 36). Thirdly, the use of FTA cards, and their analysis in facilities with high containment operating under strict biosecurity regulations have lessened biosafety concerns. Collectively, these advances now enable researchers to extend their understanding of host–vector–pathogen interactions.

In Mexico, 234 mosquito species have been recorded (37). As some (*Aedes aegypti*. *Ae. albopictus, Culex quinquefasciatus*) are key vector species, Mexico is experiencing ongoing circulation of arboviruses such as chikungunya (CHIKV), dengue (DENV), Zika (ZIKV), and West Nile (WNV) (38). Sylvatic settings in Chiapas such as the Lancadon Jungle represent much of the tropical forests in Mexico (39). Although it is one of the most biodiverse regions in Central America, it faces imminent destruction due to human activities (40). There is little information about mosquito diversity or the arboviruses circulating in the Lancadon Jungle or in other reserves in Mexico with the exception of one previous study (41). In addition, only a few epidemiological studies have investigated blood meal identification in Mexican mosquitoes. For example, (42) studied the host feeding preference of *Cx. quinquefasciatus* in Monterrey, whereas (3), (43), and (44) examined cities in the Yucatán Peninsula, or (45) within a montane forest. In this study, an integrated approach including mosquito identification using morphology and DNA barcoding, blood meal identification using high-throughput sequencing, and arbovirus screening using reverse transcriptase–polymerase chain reaction (RT-PCR) was used to characterize the mosquito fauna and unravel the host–vector–pathogen interactions in sylvan communities in Chiapas State. Furthermore, this study employs a novel method of identifying vertebrate host DNA from residual traces within arthropod RNA extracts.

## Methodology

### Study Area, Collection, and Morphological Identification of Mosquitoes

**Figure 1:**
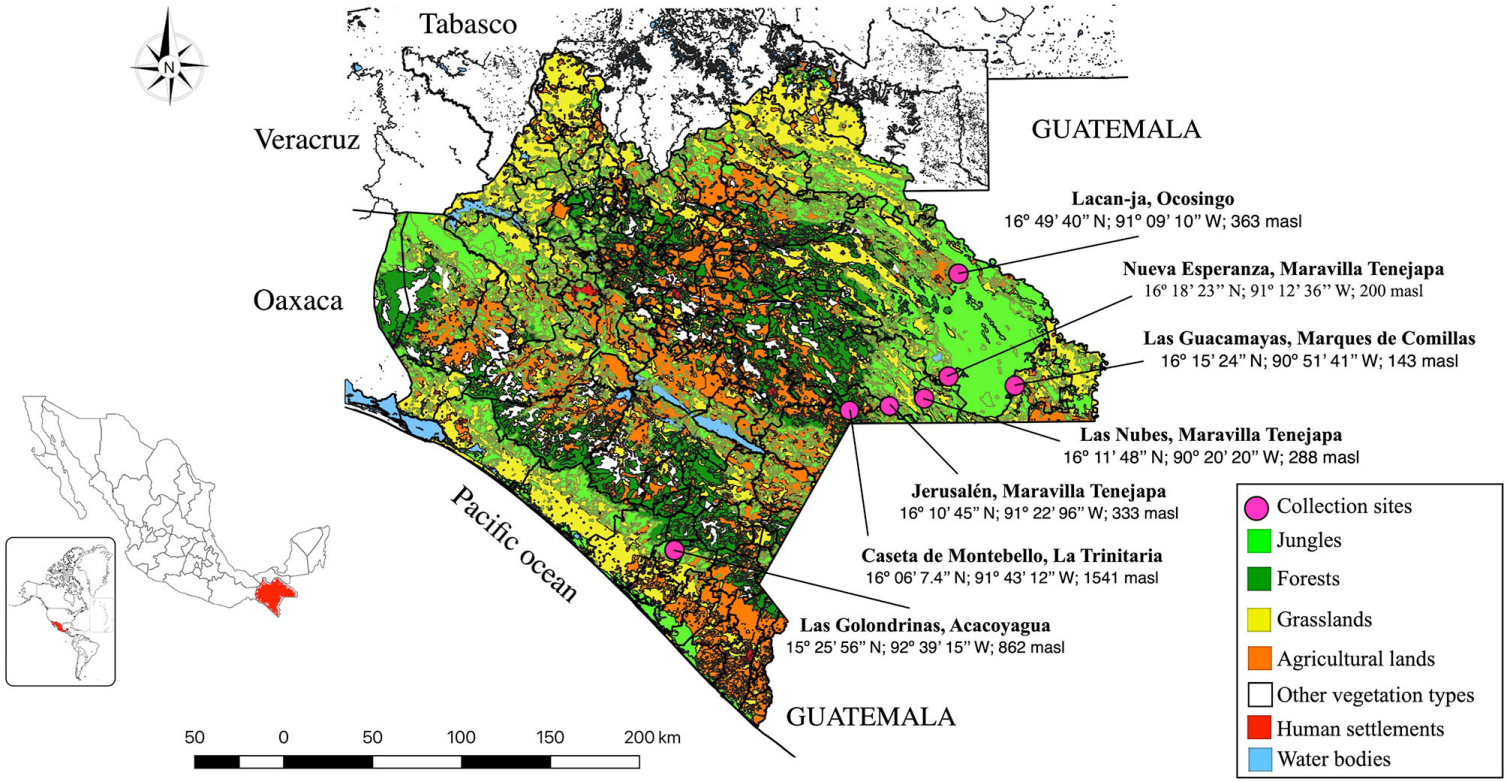
Study area showing the mosquito's collection sites of sylvan communities in Chiapas State, Mexico.

Located in southeastern Mexico, Chiapas State has an area of 73,311 km^2^ and is bordered to the north by the States of Tabasco, to the east by Guatemala, to the west by the States of Oaxaca and Veracruz, and to the south by the Pacific Ocean. The weather is tropical or subtropical and Chiapas is divided into 11 physiographic regions, seven Biosphere Reserves (BR), and three National Parks (NP). One NP (“Lagos de Montebello”) and two BR (“El Triunfo” and “Montes Azules”) were sampled in this study (Figure 1). In total, seven sylvan communities were sampled during the rainy season of July–August 2016, from the NP Lagos de Montebello (Caseta de Montebello in La Trinitaria municipality 16°06′7.4″N−91°43′12″W, 1,541 masl), from BR El Triunfo (Las Golondrinas in Acacoyagua municipality 15°25′56″N−92°39′15″W, 862 masl), and from BR Montes Azules (Las Nubes 16°11′48″N−90°20′20″W, 288 masl; Jerusalén 16°11′34.3″N−91°22′47.3″W; 333 masl; and Nueva Esperanza 16°18′23″N−91°12′36″W, 200 masl in Maravilla Tenejapa municipality; Las Guacamayas 16°15′24″N−90°51′41″W, 143 masl in Marqués de Comillas municipality, and Lacanjá 16°49′40″N−91°09′10″W, 363 masl in Ocosingo municipality) (Figure 1, Table 1). Mosquitoes were collected from inside homes and from resting places in close proximity to them. In each locality, collections were made using 10 octanol-baited CDC light traps that were deployed every 30 m following a transect at 1–1.5 m above ground level at night (18:00–22:00); the collecting effort per site was similar. Shannon traps baited with humans were also used at night (20:00–3:00), and mosquitoes were also collected from resting places using two Insectzookas (BioQuip No. 2888A) during the day between 9:00 and 17:00. In addition, immatures were collected from aquatic habitats and held alive in individual tubes to obtain adults and associated exuviae. Adults were killed using triethylamine vapors, stored in vials, and preserved in liquid nitrogen vapors. All material was transported to the Molecular Biology Laboratory, Parasitology Department Universidad Autónoma Agraria Antonio Narro, Unidad Laguna (UAAAN-UL) for taxonomic identification. In the laboratory, representatives of each species (unfed females and males when available) were pinned and identified using taxonomic keys. The classification system proposed by Wilkerson et al. (46) for the Aedini tribe and (47) for the rest of tribes and Anophelinae was followed.

**Table 1:** Checklist of mosquito species collected in seven sylvan communities in Chiapas State, Mexico.

**Species**	**Las Golondrinas**	**Caseta de Montebello**	**Las Nubes**	**Jerusalén**	**Nueva Esperanza**	**Las Guacamayas**	**Lacanjá**
**Anophelinae**							
1. *Anopheles* (*Anopheles*) *eiseni*	X	X			X		
*2. An*. (*Anopheles*) *pseudopunctipennis*					X	X	
3. *An*. (*Kerteszia*) *neivai*		X					
4. *An*. (*Nyssorhynchus*) *albimanus*					X		
**Culicinae**							
5. *Aedes* (*Georgecraigius*) *fluviatilis*			X				
6. *Ae*. (*Howardina*) *allotecnon*		X					
7. ***Ae***. **(*****Howardina*****)** guatemala^*^		X					
8. *Ae*. (*Howardina*) *quadrivittatus*	X	X					X
9. *Ae*. (*Ochlerotatus*) *angustivittatus*	X				X	X	
10. *Ae* (*Ochlerotatus*) *euplocamus*					X	X	
11. *Ae*. (*Ochlerotatus*) *fulvus*					X		
12. *Ae*. (*Ochlerotatus*) *serratus*					X	X	X
13. *Ae*. (*Ochlerotatus*) *trivitatus*					X		
14. ***Ae***. **(*****Protomacleaya*****)** insolitus^*^	X				X		
15. *Ae*. (*Protomacleaya*) *podographicus*	X	X					
16. *Ae*. (*Stegomyia*) *aegypti*			X				X
17. *Ae*. (*Stegomyia*) *albopictus*	X		X			X	
18. *Haemagogus* (*Haemagogus*) *equinus*		X					
19. *Hg*. (*Haemagogus*) *mesodentatus*					X	X	X
20. *Psorophora* (*Grabhamia*) *cingulata*					X		
21. *Ps*. (*Grabhamia*) *columbiae*					X	X	
22. *Ps*. (*Janthinosoma*) *albipes*					X	X	
23. *Ps*. (*Janthinosoma*) *champerico*					X	X	
24. *Ps*. (*Janthinosoma*) *ferox*					X	X	X
25. *Ps*. (*Psorophora*) *ciliata*						X	
26. *Culex* (*Anoedioporpa*) *restrictor*							X
27. *Cx*. (*Culex*) *coronator s.l*.					X	X	
28. *Cx*. (*Culex*) *mollis*						X	
29. *Cx*. (*Culex*) *nigripalpus*	X		X		X	X	
30. *Cx*. (*Culex*) *pinarocampa*			X				
31. *Cx*. (*Culex*) *quinquefasciatus*					X	X	X
32. *Cx*. (*Culex*) usquatus^**^			X				
33. *Cx*. (*Melanoconion*) *bastagarius*						X	
34. *Cx*. (*Melanoconion*) eastor^**^			X				
35. *Cx*. (*Melanoconion*) *erraticus*						X	
36. *Cx*. (*Melanoconion*) pedroi^**^					X		
37. *Cx*. (*Melanoconion*) *pilosus*			X			X	X
38. *Cx*. (*Melanoconion*) *spissipes*						X	
39. *Cx*. (*Microculex*) *daumastocampa*				X			
40. *Cx*. (*Microculex*) *rejector*		X	X	X			
41. *Cx*. (*Phenacomyia*) *corniger*							
42. *Mansonia* (*Mansonia*) *titillans*						X	
43. *Johnbelkinia ulopus*					X		
44. Limatus asulleptus^*^					X		
45. *Li. durhamii*			X		X	X	
46. *Sabethes* (*Sabethes*) *cyaneus*					X		
47. *Sa*. (*Sabethoides*) *chloropterus*		X					
48. *Shannoniana moralesi*	X	X			X		X
49. *Trichoprosopon digitatum*					X		
50. Tr. pallidiventer^*^					X		
51. *Tr*. nr. *brevipes*					X		
52. *Trichoprosopon* sp. nr. spG							
53. *Wyeomyia* (*Decamyia*) *pseudopecten*					X		
54. *Wy*. (*Triamyia*) aporonoma^**^	X	X			X		
55. *Wy*. (*Wyeomyia*) *abebela*	X	X	X	X	X		
56. *Wy*. (*Wyeomyia*) *adelpha/Wy. guatemala* groups I, II, III, IV	X	X	X		X	X	
57. *Wy*. (*Wyeomyia*) *melanopus*	X	X	X	X			
58. *Wy*. (*Wyeomyia) stonei*		X					
59. *Wy*. (*Wyeomyia*) sp. nr. *Wy. complosa*					X		
60. *Toxorhynchites* (*Lynchiella*) haemorrhoidalis^**^			X				
61. *Uranotaenia* (*Uranotaenia*) *lowii*				X			

* *(In bold) New national records for Mexico*.

** *New records for Chiapas State*.

Fully engorged females of identified specimens were individually placed in 1.5 Eppendorf® tubes for blood meal host detection, whereas pools of the remaining unfed adults (2–15 females and males in each pool) were placed in 1.5 mL Eppendorf® tubes for virus detection and DNA barcoding. The mounted specimens, adults on insect pins and immature stages, and exuviae mounted on microscope slides were deposited in the Culicidae Collection of the UAAAN-UL, whereas the remaining specimens in tubes were preserved on dry ice and sent to the Animal and Plant Health Agency, UK (APHA), for molecular analysis.

### DNA Extraction and Sanger Sequencing for Mosquito Molecular Identification

Standard DNA barcoding protocols (i.e., sequencing of 658 bp barcode region of COI) were used to identify unfed specimens of the morphospecies. For DNA extraction, a modified Hotshot technique (44, 48) was employed. Briefly, one to two legs from single specimens were placed directly into 50 μL of alkaline lysis buffer in a 96-well plate, which was then sonicated in a water bath for 20 min. The plate was subsequently incubated in a thermocycler for 30 min at 94°C and cooled for 5 min at 4°C, after which 50 μL of the neutralizing buffer was added to each well. PCR amplification of the full-length COI barcode region (27, 28) was performed using a protocol and primers developed by Montero-Pau et al. (LCO1490 and HCO2198) and a QIAgen PCR system with the following reaction mix, final volume 50 μL: 2 μL of DNA template, 25 μL H_2_O, 5 μL NH_4_, 5 μL of dNTPs (2 mM/μL), 2.5 μL of MgCl_2_ (25 mM/μL), 0.1 μL Bioline Taq Polymerase (Bioline Reagents Ltd., London, UK), 5 μL of each primer (each at 10 pmol/μL), and 0.38 μL of bovine serum albumin (20 mg/mL) (48, 49). The thermal profile consisted of the following: an initial denaturation step at 94°C for 1 min, 5 cycles of preamplification of 94°C for 1 min, 45°C for 1.5 min, 72°C for 1.5 min, followed by 35 cycles of amplification of 94°C for 1 min, 57°C for 1.5 min, and 72°C for 1 min, followed by a final elongation step of 72°C for 5 min. All PCR products were visualized with a 1.5% agarose gel, and samples showing bands of the correct size were bidirectionally sequenced using the ABI PRISM® BigDye® Terminator v3.1 Cycle Sequencing Kit (Applied Biosystems) at the Sequencing Unit, APHA.

### RNA Extraction and Sanger Sequencing for Blood-Fed Mosquito Molecular Identification

Blood-fed females were subjected to more extensive analysis than their unfed counterparts. Because of the potential presence of pathogens in the blood meals, RNA extraction was performed in a high-biosecurity facility at the APHA. Engorged abdomens were individually transferred from their Eppendorf storage tubes into 2 mL Qiagen flat-cap disruption tubes containing two pretreated 5-mm stainless-steel beads and 500 μL of tissue cell culture media (E-MEM/10%FBS). Each microtube was homogenized for 3 min at 25 Hz in a TissueLyser (Qiagen) and then centrifuged for 3 min at 14,000 *g*. One hundred microliters of the supernatant was removed and stored at −80°C for potential virus isolation, whereas the remainder was used for RNA purification using TRIzol following the recommended protocol (www.thermofisherscientific.com). Contrary to most RNA extraction protocols, residual co-purified DNA was not removed via DNase treatment. The RNA extracts therefore contained trace amounts of DNA from both the blood meal host and the mosquito, allowing its identification via standard barcoding.

To that end, 50 μL of RNA extract was sent to the Center for Biodiversity Genomics, at the University of Guelph for further analysis. Because of accidental loss of the cold chain during courier transportation from Mexico to APHA, which compromised DNA preservation, mosquitoes were identified using the primers AncientLepF3 (TTATAATTGGDGGWTTTGGWAATTG) and AncientLepR3 (CCTCCATGRGCRATATTWGADG), which amplify a short fragment (120–180 bp) of the COI barcode region (50). Sanger sequencing was performed following standard protocols (27, 28, 36).

### Phylogenetic Analysis of Mosquito COI Sanger Sequences

The resulting Sanger trace files from both unfed and blood-fed mosquitoes were edited and analyzed in the same manner. Paired bidirectional traces were combined to produce a single consensus sequence for the full 658-bp barcode sequence for the unfed mosquitoes and a shorter 130-bp barcode sequence for the blood-fed mosquitoes. For species recorded in the collecting sites, but from which we could not obtain a DNA barcode sequence, we employed sequences from the Barcode of Life Database (BOLD-www.barcodingoflife.org) or NCBI (www.ncbi.nlm.nih.gov/genbank/). In total, 20 species and 139 sequences were added to the dataset (Supplementary Table 1); no sequences of *An. neivai, Cx. bastagarius, Cx. daumastocampa, Cx. spissipes*, or *Wy. stonei* were included in the analysis.

Genetic relationships between species were analyzed using three methods: neighbor joining (NJ), maximum likelihood (ML), and maximum parsimony (MP). For the NJ and MP, the dataset was analyzed in MEGA v.6 (51). NJ analysis employed the K2P distance metric. Bootstrap values to test the robustness of the tree were obtained by conducting 1,000 pseudoreplicates; only groups with more than 80% bootstrap support are shown (19, 52). The MP tree was obtained using the subtree–pruning–regrafting algorithm with the initial trees obtained by the random addition of sequences (10 replicates). ML analysis was implemented in PhyML 3.0 (52); branch support was calculated using approximate likelihood ratio tests (53). For the phylogenetic analyses, a COI DNA barcode sequence of a black fly, *Simulium weji* Takaoka (accession no. KF289451) was used as an outgroup. NJ, MP, and ML trees were exported as JPG files in Acrobat 8.Professional, and then Adobe Photoshop CS3 (v. 10.0.1) was used to edit them.

After sequences were uploaded to BOLD, most barcode sequences longer than 500 bp were assigned a Barcode Index Number (BIN), a taxonomic system that assigns similar barcode sequences into species proxies without the need for Linnaean nomenclature (54). An NJ tree composed of BINs was generated on BOLD, and each morphospecies was mapped to BINs in the tree. Taxonomic discordance in our dataset was analyzed using BOLD tools, one of which provides a means of confirming the concordance between barcode sequence clusters and species designations.

### Next-Generation Sequencing of Blood-Fed Female Mosquitoes for Host Identification

The same RNA extracts employed for mosquito identification were used for blood meal identification via next-generation sequencing (NGS). As mentioned previously, following RNA extraction, residual DNA was not removed by DNase treatment.

Instead, the residual DNA was used as a template for PCR. A two-step PCR protocol was used to amplify blood meal (host) DNA and to prepare it for sequencing on an Ion Torrent platform. The first PCR reaction consisted of 6.25 μL of 10% d-(+)-trehalose dihydrate (Fluka Analytical), 2.0 μL of Hyclone ultra-pure water (Thermo Scientific), 1.25 μL of 10X PlatinumTaq buffer (Invitrogen), 0.625 μL of 50 mM MgCl^2^ (Invitrogen), 0.125 μL of each 10 μM primer cocktail, 0.0625 μL of 10 mM dNTP (KAPA Biosystems), 0.060 μL of 5U/lL PlatinumTaq DNA Polymerase (Invitrogen), and 2 μL of RNA, for a total reaction volume of 12.5 μL. The primers (BloodmealF1_t1, BloodmealF2_t1, VR1_t1, VR1d_t1, and VR1i_t1; Table 2) were designed to amplify a 185-bp region of the COI barcode from diverse birds and mammals and were tailed with M13F and M13R sequences that provided universal primer binding sites during the second round of PCR. Thermocycling consisted of an initial denaturation at 95°C for 2 min, 60 cycles of 95°C for 40 s, 56°C for 40 s, and 72°C for 30 s, and a final extension at 72°C for 5 min. After PCR, the products were visualized on a 2% E-gel (Invitrogen) to confirm amplification and were then diluted 2-fold with sterile water.

**Table 2:** Vertebrate-specific primers used for first-round PCR from blood-fed mosquito's species collected in sylvan communities in Chiapas State, Mexico.

**Primer Name**	**Sequence (5^**′**^->3^**′**^)**	**Direction**	**References**
BloodmealF1_t1	**TGTAAAACGACGGCCAGT**ACCACWATTATTAAYATAAARCCMC	Forward	(55, 56), This study.
BloodmealF2_t1	**TGTAAAACGACGGCCAGT**ACTACAGCAATTAACATAAAACCMC	Forward	(55, 56), This study.
VR1_t1	**CAGGAAACAGCTATGAC**TAGACTTCTGGGTGGCCAAAGAATCA	Forward	(24)
VR1d_t1	**CAGGAAACAGCTATGAC**TAGACTTCTGGGTGGCCRAARAAYCA	Reverse	(24)
VR1i_t1	**CAGGAAACAGCTATGAC**TAGACTTCTGGGTGICCIAAIAAICA	Reverse	(24)

*The sequences of the M13F and M13R tails are in bold, whereas the COI-specific sequences are in regular font. The M13 tails served as second-round PCR primer binding sites, on which Ion Torrent sequencing adapters and UMIs (IonXpress 1–96) were fused*.

The diluted products were then used as template for a second round of PCR using M13F primers tailed with IonXpress universal molecular identifiers (UMIs) tags and the Ion Torrent “A” sequencing adapter, and M13R primers tailed with the Ion Torrent trP1 sequencing adapter (Table 1 for primer sequences). Reaction components for the second round of PCR were identical to the first; the thermocycling regimen consisted of an initial denaturation at 95°C for 2 min, 5 cycles of 95°C for 40 s, 45°C for 40 s, and 72°C for 30 s, 35 cycles of 95°C for 40 s, 51°C for 40 s, and 72°C for 30 s, and a final extension at 72°C for 5 min. The products of the second round of PCR were pooled in equal volumes and purified by mixing 400 μL of pooled product with 200 μL of purification beads (Aline Biosciences, Woburn, MA, USA) for a ratio of 0.5X beads: DNA (vol:vol). The mixture was incubated at room temperature for 8 min to allow the DNA to bind to the beads, after which the beads were pelleted on a magnetic rack. The supernatant (550 μL) was transferred to a clean 1.5-mL tube and mixed with 113 μL of sterile water and 417 μL of fresh purification beads for a final ratio of 1.2X beads: DNA (vol:vol). The mixture was incubated at room temperature for 8 min and then pelleted on a magnet. The supernatant was carefully discarded, and the pellet was washed three times with 1 mL of freshly prepared 80% ethanol and then air-dried. The purified product was eluted from the beads by resuspending them in 200 μL of sterile water, pelleting the beads, and then carefully transferring 180 μL of the supernatant to a clean tube. The purified product was quantified using a Qubit 2.0 fluorometer and adjusted to 22 pM with sterile water. The 22 pM library was then sequenced on an Ion Torrent PGM following the manufacturer's instructions using a 316v.2 chip. Each sequence was automatically assigned to its source sample via the UMI tags by the Torrent Browser suite.

The raw reads for each sample were then processed through a custom analytical pipeline that first filtered the reads based on a minimum quality value (PHRED = 20) and a minimum read length of 100 bp. All adapter and primer sequences were identified and removed using CutAdapt (57). As the forward primer should be readily visible in the reads, those lacking it were discarded, so only high-quality reads were included in the final dataset. The trimmed reads were collapsed into unique haplotypes (http://hannonlab.cshl. edu/fastx_toolkit/index.html) while retaining the original read counts. Each sequence was used to query (BLAST) a custom database composed of global vertebrate COI sequences downloaded from BOLD. The resulting BLAST hits were filtered to retain only those with a minimum match of 95% identity and 100 bp of coverage between the queried sequence and a reference sequence. Furthermore, identifications were retained only if supported by at least 50 original reads.

### Virus Testing

Virus screening was performed on all blood-fed specimens that were analyzed for host DNA, as well as on pools of adult mosquitoes that were not previously analyzed (the former provided a detailed screen at the individual level, whereas the latter screened at the population level). In the case of the pools, each morphospecies was separated into subsets containing 2–15 specimens per tube, and the same methodology employed for homogenizing the engorged abdomens was followed. Again, 100 μL of the homogenate was stored at −80°C for potential virus isolation, whereas the remainder was used for RNA extraction using TRIzol (www.thermofisherscientific.com).

The RNA samples were screened for the presence of common viruses using a one-step semiquantitative SYBR Green RT-PCR employing generic primers that target a broad range of *Flavivirus* and *Alphavirus* species. For *Flavivirus* detection, we used the following primers of Johnson et al. (58): Flavi Forward (GTRTCCCAKCCDGCNGTRTC) and Flavi Reverse (GCMATHTGGTWCATGTGG). The primers of Johnson et al. (58) were used for *Alphavirus* detection: VIR2052 Forward (TGGCGCTAGATGAAATCTGGAATGTT) and VIR2052 reverse (TACGTGTTGTCGTCGCCG ATGAA). The RT-PCR reactions included 6.25 μL of molecular grade water, 12.5 μL of QuantiTect SYBR Green RT-PCR kit (Qiagen), 0.25 μL of Quantitec RT Mix (Qiagen), 2 μL of each primer at 10 pmol/μL, and 2 μL of RNA. Thermocycling consisted of one cycle of reverse transcription at 50°C for 30 min, one cycle of initial denaturation at 95°C for 15 min, 45 cycles of amplification at 95°C for 15 s, 60°C for 30 s, and 72°C for 30 s, and a final cycle of 95°C for 1 min, 55°C for 30 s, and 95°C for 30 s. RNA from the WNV (Goose Israel strain) and the Sindbis virus (SINDV) (Germany 5.3 strain) was used as positive controls for flaviviruses and alphaviruses, respectively. All positive controls were passaged two or three times in Vero cells.

## Results

### Faunistic Survey and Mosquito Species Identification Using COI DNA Barcoding

The 1,634 collected specimens included representatives of two subfamilies, 14 genera, 25 subgenera, and 61 named species, as well as two taxa that could only be assigned to a genus (*Trichoprosopon, Wyeomyia*) (Table 2). The genera *Aedes* and *Culex* were the most diverse with 13 and 16 species, respectively, followed by *Psorophora* with six species. *Aedes guatemala, Ae. insolitus, Limatus asulleptus*, and *Tr. pallidiventer* represent new records for Mexico, whereas two apparently undescribed species of *Trichoprosopon* were discovered. As well, four species (*Culex usquatus, Cx. eastor, Cx. pedroi, Wy. aporonoma*, and *Tx. haemorrhoidalis*) are new records for Chiapas State. The largest number of species was collected at Nueva Esperanza (32) followed by Las Guacamayas (21), Caseta de Montebello (5), Las Nubes (14), Las Golondrinas (12), Lacanja (9), and Jerusalén (5).

In total, 570 specimens were DNA barcoded. Among these, 285 non-engorged specimens were analyzed using DNA extracted from a single leg, whereas 285 blood-fed females were analyzed using a modified protocol that employed RNA extracts as the template for DNA barcoding. The overall sequencing success was 76% (436/570) with barcodes recovered from five morphospecies, but sequences were recovered from 96% (273/285) of the DNA extracts from a single leg with most (235) >300 bp in length. By contrast, only 38% (108/285) of the blood-fed specimens yielded a sequence >130 bp in length (Supplementary Data Sheet 1).

**Figure 2:**
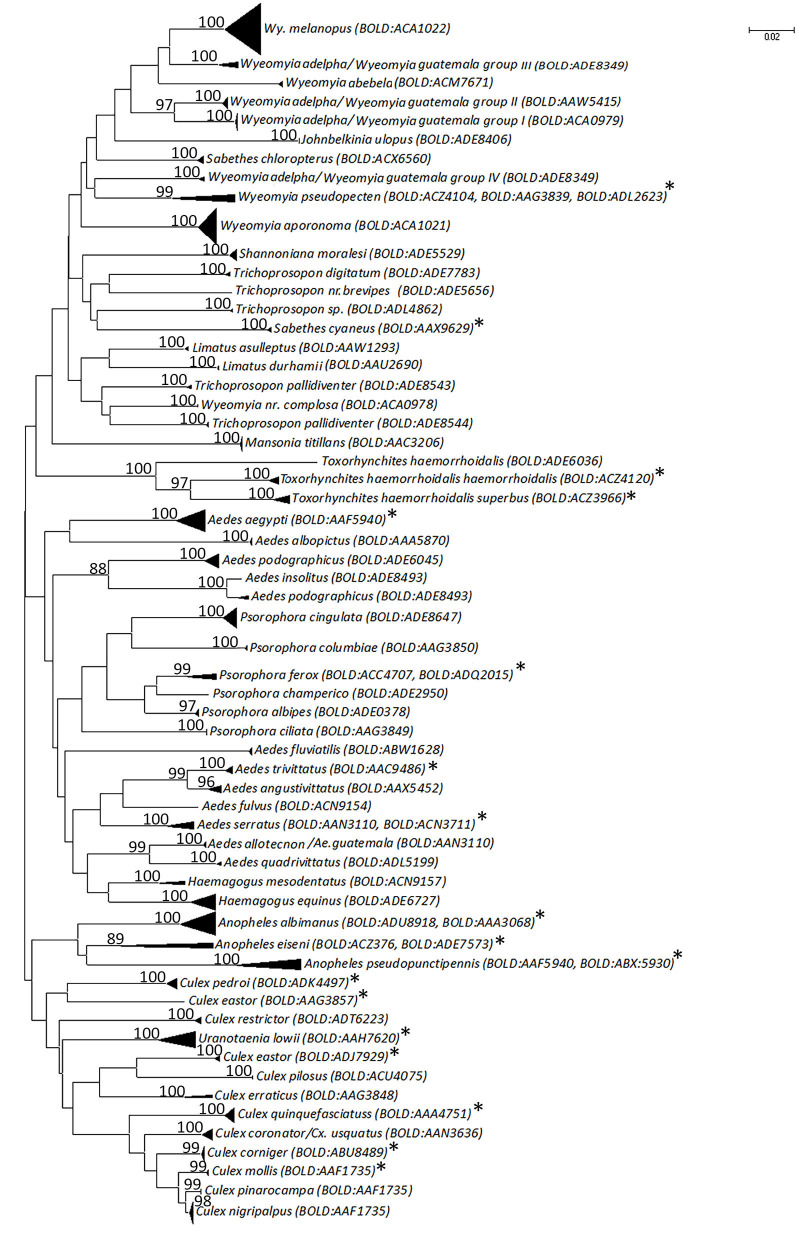
Neighbor-joining tree based on the the Kimura two-parameter distances of *COI* DNA barcodes (>300 bp) for mosquito species recorded in sylvan communities in Chiapas State, Mexico. A divergence > 2% may be indicative of separate operational taxonomic units. Only bootstrap support values > 80% are shown. An asterisk (*) relates to species from which sequences have been downloaded from BOLD and NCBI databases.

The 291 sequences of >300 bp in length were combined with 78 publicly available sequences from BOLD from Mexico and other countries in the Americas (representing 20 species) to create a final dataset with 369 sequences. Intraspecific sequence divergences were variable across taxa, ranging from zero to 7.30% with an average of 1.56% (Table 3). Because the NJ, ML, and MP trees had similar topology and strong support values, only the NJ tree (Figure 2) is shown (see Supplementary Figures 1, 2 for ML and MP trees, respectively). High intraspecific K2P distance (above 2%) was observed for nine taxa: *Anopheles eiseni*—average of 5.39% (maximum of 7.76% among three specimens), *An. pseudopunctipennis*—average of 2.79% (maximum of 5.4% among seven specimens), *Ae. podographicus*—average of 4.05% (maximum of 11.45% among 11 specimens), *Cx. eastor*—average 4.88% (maximum of 15.8% among six specimens), *Cx. erraticus*–average 2.28% (maximum of 2.28% between two specimens), *Tr. pallidiventer*—average of 4.95% (maximum of 8.2% among four specimens), *Wy. adelpha*/*Wy. guatemala*—average of 7.30% (maximum of 12.14% among 27 specimens), *Wy. pseudopecten*—average 4.05% (maximum of 11.96% among five specimens), and *Tx. haemorrhoidalis*—average of 4.30% (maximum 12.71% among 11 specimens). Interspecific divergence values were low for a few species such as *Cx. nigripalpus/Cx mollis* (1.84%) and *Cx. nigripalpus/Cx. quinquefasciatus* (6.7%), but much higher between species in different genera such as *Tx. haemorroidalis/Cx. quinquefasciatus* (20.62%) and *An. pseudopunctipennis/Sa. cyaneus* (21.81%) (Supplementary Table 2).

**Table 3:** List of mosquito species and number of specimens (*n*) from which DNA barcodes (>400 bp) were obtained collected at sylvan communities in Chiapas State, Mexico.

**Species**	**Average genetic diversity (%)**	**Country**	***n***	**BOLD BIN**
**Anophelinae**				
*Anopheles albimanus*	1.47%	Colombia, Mexico	16	BOLD:ADU8918
*Anopheles eiseni*	5.39%	French Guiana, Mexico	3	BOLD:ACZ3766, BOLD:ADE7573
Anopheles pseudopunctipennis^*^	2.79%	Colombia, Mexico	7	BOLD:ABX5930, BOLD:AAF5940
**Culicinae**				
*Aedes albopictus*	0.10%	Mexico	5	BOLD:AAA5870
*Aedes alloctenon*	0.06%	Mexico	3	BOLD:ACT1072
*Aedes angustivitatus*	0.88%	Mexico	5	BOLD:AAX5452
*Aedes aegypti*	1.48%	Mexico, Puerto Rico, USA	15	BOLD:AAF5940
*Aedes fluviatilis*	0.10%	Mexico	1	BOLD:ABW1628
*Aedes fulvus*	n/a	Mexico	1	BOLD:ACN9154
*Aedes guatemala*	n/a	Mexico	1	BOLD:ACT1072
*Aedes insolitus*	n/a	Mexico	1	BOLD:ADE8493
Aedes podographicus^*^	4.05%	Mexico	11	BOLD:ADE6045, BOLD:ADE8493
*Aedes quadrivittatus*	0.32%	Mexico	2	BOLD:ADL5199
*Aedes serratus*	1.68%	French Guiana, Mexico	4	BOLD:AAN3110, BOLD:ACN3711
*Aedes trivittatus*	0.46%	Canada	5	BOLD:AAC9486
*Culex corniger*	0.12%	Colombia	10	BOLD:ABU8489
*Culex coronator* s.l. ^**^	0.60%	Mexico	6	BOLD:AAN3636
Culex eastor^*^	4.88%	Brazil, Mexico	5	BOLD:AAG3857, BOLD:ADJ7929
*Culex erraticus*	2.28%	Mexico	2	BOLD:AAG3848
*Culex quinquefasciatus*	0.12%	Brazil, French Guiana, USA	10	BOLD:AAA4751
*Culex mollis*	0.08%	Brazil	4	BOLD:AAF1735
*Culex nigripalpus*	0.17%	Mexico	15	BOLD:AAF1735
*Culex pedroi*	0.67%	Brazil	7	BOLD:ADK4497
*Culex pinarocampa*	0%	Mexico	3	BOLD:AAF1735
*Culex pilosus*	0%	Mexico	2	BOLD:ACU4075
*Culex restrictor*	0.25%	Mexico	4	BOLD:ADT6223
Culex usquatus^**^	0.48%	Mexico	2	BOLD:AAN3636
*Haemagogus equinus*	1.13%	Mexico	11	BOLD:ADE6727
*Haemagogus mesodentatus*	2.11%	Mexico	2	BOLD:ACN9157
*Johnbelkinia ulopus*	0%	Mexico	3	BOLD:ADE8406
*Limatus asulleptus*	0.21%	Mexico	3	BOD:AAW1293
*Limatus durhamii*	0.11%	Mexico	3	BOLD:AAU2690
*Mansonia titillans*	0.03%	Mexico	10	BOLD:AAC3206
*Psorophora albipes*	0.19%	Mexico	5	BOLD:ADE0378
*Psorophora champerico*	n/a	Mexico	1	BOLD:ADE2950
*Psorophora ciliata*	0%	Mexico	4	BOLD:AAG3849
*Psorophora cingulata*	0.46%	Mexico	14	BOLD:ADE8647
*Psorophora columbiae*	0.38%	Mexico, USA	7	BOLD:AAG3850
*Psorophora ferox*	1.49%	Mexico, USA	4	BOLD:ADQ2015, BOLD:ACC4707
*Sabethes chloropterus*	0.55%	Mexico	5	BOLD:ACX6560
*Sabethes cyaneus*	0.16%	Colombia, USA	3	BOLD:AAX9629
*Shannoniana moralesi*	0.39%	Mexico	8	BOLD:ADE5529
Toxorhynchites haemorrhoidalis (sub. haemorrhoidalis, sub. superbus)^*^	4.35%	French Guiana, Mexico	11	BOLD:ADE6036, BOLD:ACZ4120/BOLD:ACZ3996, BOLD:ACZ3913
*Trichoprosopron* nr. *brevipes*	0%	Mexico	1	BOLD:ADE5656
*Trichoprosopron digitatum*	0.21%	Mexico	3	BOLD:ADE7783
Trichoprosopron pallidiventer^*^	4.95%	Mexico	5	BOLD:ADE8543, BOLD:ADE8544
*Trichoprosopon* sp. nr. *Tr*. stG	0.16%	Mexico	2	BOLD:ADL4862
*Uranotaenia lowii*	1.53	Mexico, Puerto Rico, USA	11	BOLD:AAA7620
*Wyeomyia abebela*	0.24%	Mexico	4	BOLD:ACA1022
*Wyeomyia adelpha/Wy. guatemala* groups I, II, III, IV^*^	7.30%	Mexico	27	BOLD:ACA0979 (group I), BOLD:AAW5415 (group II), BOLD:ADE:8349 (groups III, IV)
*Wyeomyia aponoroma*	0.29%	Mexico	25	BOLD:ACA1021
*Wyeomyia melanopus*	1.42%	Mexico	35	BOLD:ACM7671
Wyeomyia pseudopecten^*^	4.04	French Guiana, Mexico	5	BOLD:ADL2623, BOLD:ACZ4104, BOLD:AAG3839
*Wyeomyia* nr. *complosa*	0%	Mexico	2	BOLD:ACA09978

* *Taxa with > 2% genetic divergence*.

** *Taxa with same BIN*.

*Mean (%) intraspecific values of sequence divergence (Kimura 2-parameter distance) are shown with missing entries, indicating that less than two barcode sequences were obtained*.

NJ analysis showed that most conspecific specimens formed a single cluster in the tree with high bootstrap support value (Figure 2), but there were exceptions. *Ae. podographicus* split into two groups that were assigned to different BINs (BOLD:ADE8493, BOLD:ADE6045). Likewise, specimens of *Cx. eastor* were assigned to two BINs (BOLD:AAG3857, BOLD:ADJ7929). *Trichoprosopon pallidiventer* was similarly divided into two BINs (BOLD:ADE8543, BOLD:ACA0979), whereas *Wy. adelpha*/*Wy. guatemala* showed a deep division in the NJ tree forming four groups, here designated as group I (BOLD:ACA0979), group II (BOLD:AAW545), group III, and group IV both with BINs number (BOLD:ADE:8349), each supported with 100% bootstrap values (Figure 2). Interestingly, specimens of *Tr. haemorrhoidalis* from Mexico (BOLD:ADE6036) clustered separately from their French Guiana counterparts: *Tx. haemorrhoidalis haemorrhoidalis* (BOLD:ACZ4120) and *Tx. haemorrhoidalis superbus* (BOLD: ACZ3966). By contrast, two pairs of morphologically identified species (*Ae. alloctenon* + *Ae. guatemala, Cx. coronator* + *Cx. usquatus*) showed intermingling of their barcodes (Figure 2). The BOLD ID engine was used to identify specimens that lacked a species assignment based on morphological study. Two sequences assigned to *Wyeomyia* sp. 98.3% similarity to Costa Rican *Wy. complosa* (BOLD:ACA0978), so they were assigned to this species.

The 369 barcode sequences generated in this study represented 64 BINs deriving from 55 morphologically identified species. Of these, 42 were represented by a single BIN, seven were represented by two, whereas three BINs were recognized in *Wy. adelpha/Wy. guatemala* and *Wy. pseudopecten*, and four within *Tx. haemorrhoidalis* (Figure 2, Table 3). Eight species shared a BIN with at least one other species in its genus. Most of these cases involved species of *Aedes* (*Ae. allotecnon, Ae. guatemala, Ae. insolitus*, and *Ae. podographicus*) or *Culex* (*Cx. coronator, Cx. mollis, Cx. nigripalpus, Cx. pinarocampa*, and *Cx. usquatus*) (Figure 2, Table 3).

### Identification of Vertebrate Hosts From Mosquito Blood Meals

The 285 females collected with varying degrees of blood engorgement in the Sella scale represented 22 morphospecies (Table 4). The source of the blood meal was ascertained for 30% (59) of these mosquitoes. They included representatives from three genera and eight species: *Ae. angustivittatus, Ae. podographicus, Ae. trivittatus, Cx. quinquefasciatus, Cx. nigripalpus, Culex* sp., *Ps. albipes*, and *Ps. ferox* (Table 4). The others failed to generate host information despite repeated attempts at PCR.

**Table 4:** Checklist of blood-fed mosquito's species and total number of specimens (*n*) collected in sylvan communities in Chiapas State, Mexico.

**Species**	***n***
**Anophelinae**	
*Anopheles pseudopunctipennis*	3
**Culicinae**	
*Aedes aegypti*	7
*Aedes albopictus*	4
*Aedes angustivittatus*	14
*Aedes podographicus*	1
*Aedes serratus*	3
*Aedes* sp.	1
*Aedes trivittatus*	2
*Culex bastagarius*	2
*Culex corniger*	1
*Culex coronator* s.l.	1
*Culex erraticus*	1
*Culex nigripalpus*	5
*Culex pedroi*	1
*Culex pilosus*	4
*Culex quinquefasciatus*	201
*Culex* sp.	5
*Culex spissipes*	1
*Limatus durhamii*	5
*Mansonia titillans*	1
*Psorophora albipes*	6
*Psorophora champerico*	3
*Psorophora columbiae*	1
*Psorophora ferox*	6
*Sabethes chloropterus*	1
*Uranotaenia lowii*	1
*Wyeomyia adelpha/Wy. guatemala*	4

Analysis of the 80 vertebrate sequences recovered from blood meals revealed that most mosquito species fed on birds, primarily chicken (*Gallus gallus*), followed by mammals such as the Virginia opossum (*Didelphis virginiana*) and human (*Homo sapiens*) (Table 5). *Culex quinquefasciatus* showed the highest diversity in host use as it fed on chicken, turkey (*Meleagris gallopavo*), Muscovy duck (*Cairina mochata*), Great-tailed grackle (*Quiscalus mexicanus*), horse (*Equus ferus*), and cow (*Bos taurus*). To the best of our knowledge, the hosts of four species (*Ae*. *angustivittatus, Ae. podographicus, Ae. insolitus, Ae. trivittatus*) were previously unknown in Mexico.

**Table 5:** Vertebrate hosts species, host scientific and common name, and number of specimens (*n*) identified for each mosquito species collected in sylvan communities in Chiapas State, Mexico.

**Mosquito species**	**Host**	**Host common name**	**Host group**	***n***
*Aedes angustivittatus*	*Anas platyrhynchos*	Mallard	Birds	1
	*Gallus gallus*	Chicken	Birds	1
*Aedes podographicus*	*Bos taurus*	Cow	Mammals	1
*Aedes trivittatus*	*Equus ferus caballus*	Horse	Mammals	1
*Culex quinquefasciatus*	*Bos taurus*	Cow	Mammals	
	*Didelphis virginiana*	Virginia opossum	Mammals	2
	*Equus ferus caballus*	Horse	Mammals	1
	*Meleagris gallopavo*	Wild turkey	Birds	1
	*Quiscalus mexicanus*	Great-tailed grackle	Birds	1
	*Gallus gallus*	Chicken	Birds	64
	*Cairina mochata*	Muscovy duck	Birds	1
*Culex nigripalpus*	*Sus scrofa*	Wild boar	Mammals	1
*Culex* sp.	*Gallus gallus*	Chicken	Birds	1
	*Turdus grayi*	Clay-colored thrush	Birds	1
*Psorophora albipes*	*Home sapiens*	Human	Mammals	2
*Psorophora ferox*	*Bos taurus*	Cow	Mammals	1

### Virus Testing

In total, 270 blood-fed specimens and 204 pools of mosquitoes (1,064 specimens) were screened for flavivirus and alphavirus RNA (Tables 4, 6) spanning across all seven sylvan communities. No *Flavivirus* or *Alphavirus* RNA was detected in any sample. Positive controls generated expected results indicating that the assays were effective.

**Table 6:** Mosquito species, number of pools, and total number of specimens per pool (*n*) per community processed for the detection of *Flavivirus* and *Alphavirus* RNA in pools of unfed mosquitoes collected in sylvan communities in Chiapas State, Mexico.

**Species**	**Locality/no. of pools (*****n*****)**				
	**Las Golondrinas**	**Las Guacamayas**	**Las Nubes**	**Nueva Esperanza**	**Lacan-ja**
**Culicinae**					
*Aedes aegypti*			4 (10)		1 (2)
*Aedes albopictus*		1 (2)			
*Aedes angustivittatus*	1 (6)		1 (10)		23 (195)
*Aedes podographicus*	1 (8)				
*Aedes serratus*					4 (33)
*Aedes trivittatus*			1 (2)		
*Aedes* sp.	1 (5)				
*Culex quinquefasciatus*		46 (311)	14 (93)	28 (196)	19 (73)
*Culex nigripalpus*	14 (133)		1 (2)		3 (18)
*Culex pilosus*		1 (2)			
*Haemagogus* sp.	1 (6)				
*Limatus asulleptus*					1 (10)
*Limatus durhami*	1 (2)			1 (2)	
*Psorophora albipes*					1 (3)
*Psorophora champerico*		1 (2)			2 (8)
*Psorophora ferox*	1 (96)				8 (60)
*Psorophora cingulata*					5 (24)
*Sabethes* sp.	1 (8)				
*Shannoniana moralesi*					1(3)
*Wyeomyia adelpha/Wy. guatemala*	14 (117)				2 (12)

## Discussion

The elucidation of vector–host–pathogen interactions typically require separate analytical pathways: DNA for the insect vector and the vertebrate host(s), and RNA for alphaviral and flaviviral pathogens. In this study, we used morphologically identified mosquitoes from communities in Chiapas State to demonstrate that these interactions can be revealed by analyzing DNA recovered by a standard RNA extraction protocol. By eliminating the need for a separate DNA extraction, vector–host–pathogen interactions can be ascertained in a simpler, cost-effective manner, an important consideration for areas where mosquitoes vector and viral diseases occur. Among the 61 mosquito taxa detected in this study, at least 10 (*An. albimanus, An. pseudopunctipennis, Ae. albopictus, Ae. angustivittatus, Ae. aegypti, Cx. nigripalpus, Cx. quinquefasciatus, Cx. restuans, Cs. inornata, Ps. ferox*) are pathogen vectors in Mexico and other countries in the neotropics. Given the medical importance of the viruses that they transmit (60–63), the need for regular vector surveillance to aid disease control is essential in Mexico and throughout Central America.

DNA barcoding proved effective at identifying mosquito species in Quintana Roo State, Mexico (60, 61, 64), and 96% for mosquitoes processed with standard barcoding methods in this study showed similar performance. Success in barcode recovery was substantially lower (38%) for blood-fed females using residual DNA in RNA extracts as template. Residual DNA is typically removed during conventional RNA extraction, but the quantity of residual DNA likely varies with different RNA extraction methods, but this matter has not been investigated in detail so further studies should examine multiple RNA extraction methods and vector taxa to optimize DNA retention. Second, during transfer from Mexico to the UK, the blood-fed specimens were exposed to room temperatures for 48 h. Because nucleic acid degradation likely occurred, a shorter than normal barcode sequence was targeted (130 bp) for amplification. While this approach likely resulted in a higher success rate than if standard primers (e.g., 658 bp) were used, sequence recovery would certainly have increased if the specimens were frozen during transfer. Methods are available to recover longer sequences from highly degraded samples (50), but this study aimed to develop a simple approach to delineate vector–host–pathogen interactions. Despite the lower sequence recovery from blood-fed specimens, the barcodes that were recovered did confirm morphological identifications in all cases (Supplementary Data Sheet 1).

This study did not aim to examine phylogeny relationships, but the dataset was analyzed using ML and MP phylogenetic methods. Analysis of the barcode sequences with NJ (Figure 2) in comparison with ML and MP phylogenetic algorithms (Supplementary Figures 1, 2) showed fair concordance with phylogenetic relationships proposed for Anophelinae, Aeidini, Culicini, and Sabethini (5, 50, 65), confirming the phylogenetic signal present in COI (23). Intraspecific genetic divergences for most species were within the 2% limit standard for insects and the Culicidae [e.g., (27, 28, 32, 66–68)], with the exception of *An. eiseni, An. pseudopunctipennis, Ae. podographicus, Cx. eastor, Cx. erraticus, Tr. pallidiventer, Tx. haemorrhoidalis, Wy. adelpha/Wy. guatemala*, and *Wy. pseudopecten* (Table 3), whose higher intraspecific divergences and deep splits in the NJ tree (Figure 2) suggest cryptic diversity. However, future studies should use rapid mutating markers (microsatellites, Single Nucleotide Polymorphisms) to analyse more thoroughly such intraspecific diversity.

The genus *Anopheles* includes many vector species for malaria of which several are species complexes (5). Indeed, the separation of both *An. eiseni* and *An. pseudopunctipennis* into multiple BINs reveals the likely presence of cryptic lineages within them. The deep genetic divergence observed in *An. pseudopunctipennis* reinforces earlier reports that it is a species complex (69, 70). Similar cases have been documented in *An. apicimacula* and in the *An. crucians s.l*. and *An. lindesayi s.l*. complexes using DNA barcoding (71, 72). *Aedes podographicus* is a member of the Terrens group in the subgenus *Protomacleaya*, which includes some 28 nominal species and two forms with neotropical distributions (72, 73). Among these, 10 species are found in Mexico and three in Chiapas State. The adults of several of these species are so morphologically similar that their discrimination is difficult. Further morphological and zoogeographical evidence discussed in Schick (73) and Schick (74) supports the hypothesis that *Ae. podographicus* is a species complex. Another member of the Terrens group encountered in Chiapas is *Ae. insolitus*, which is also a suspected species complex related to the *Ae. podographicus* complex (73, 74).

The subgenus *Melanoconion* of *Culex* includes ~160 described species (5), making it one of the most species-rich subgenera within the Culicidae. Further taxonomic clarity is important as its members are vectors for viruses such as Venezuelan Equine Encephalitis (VEE) (74). The usefulness of DNA barcodes for discriminating species in this subgenus has been reported, as well as the discovery of cryptic species or new species within *Culex* (71, 75, 76). In this study, six species of *Melanoconion* were detected (Table 2). One of these species, *Cx. eastor*, was separated into two groups with an average genetic divergence of 4.88%, one from Mexico (BIN:AAG3857) and the other from Brazil (BIN:ADJ7929), supporting the presence of two cryptic species.

The genus *Trichoprosopon* includes 13 species in Central and South America, but their importance as disease vectors is poorly known (77). Two of these species (*Tr. digitatum, Tr. soaresi*) have been reported from Mexico (78). The present study extends this list by three species: *Tr. pallidiventer*, and a species that is close to *Tr. brevipes* from Brazil based upon morphological features (79, 80), and another undescribed taxon close to the *Trichoprosopon* spG of Talaga et al. (34). An average intraspecific diversity of 4.95% was obtained for *Tr. pallidiventer* and this group separated from other specimens identified *Tr. digitatum, Tr*. nr. *brevipes*, and *Trichoprosopon* sp. in the NJ tree (Figure 2) with high support. This suggests that the specimens identified as *Tr. pallidiventer* include two lineages, a result also noted by studies in French Guiana (34). These results support (77) conclusions that species of the genera *Runchomyia, Shannoniana*, and *Trichoprosopon* are difficult to identify because of lack of adequate descriptions. A single sequence was obtained for a specimen identified as *Trichoprosopon* nr. *brevipes*, but any final assessment of its taxonomic status requires more specimens.

Although taxonomic revision is required, the genus *Wyeomyia* includes 139 species with neotropical and Nearctic distributions (5, 81), and 10 of these species occur in Mexico (59). *Wyeomyia pseudopecten*, a member of the subgenus *Decamyia*, includes records from Guatemala, Honduras, and the Caribbean to Brazil (82). Little is known about its biology (34), but the presence of two BINs suggests it is a species complex. Specimens identified as *Wy. adelpha*/*Wy. guatemala* showed high intraspecific divergence (7.30%, *n* = 10), and barcode analysis revealed four groups, named here groups I, II, III, and IV (Figure 2), again suggesting cryptic species. Taxonomy uncertainty surrounds three species: *Wy. adelpha, Wy. guatemala*, and *Wy. mitchellii*. *Wyeomyia guatemala* was described from Guatemala [(83), p. 139], *Wy. adelpha* from Costa Rica [(82), p. 140]] and *Wy. mitchellii* from Jamaica (84). *Wyeomyia guatemala* was separated from *Wy. mitchellii* by Theobald (84) based on the morphology of the larva and the male genitalia, but the females were separated based on their geographical distribution restricting the name *Wy. guatemala* for Central America and *Wy. mitchellii* for Florida, USA, and the West Indies. However, (85, 86) placed *Wy. guatemala* as a synonym of *Wy. mitchellii*, but (87) stated that specimens from Central America identified as *Wy. guatemala* or *Wy. mitchellii* should be named as *Wy. adelpha*. This was confirmed by Belkin et al. (88) in their review of mosquitoes in Jamaica, where they concluded that supposed records of *Wy. mitchellii* from Mexico to Panama were likely to represent another species. Currently, *Wy. mitchellii* is only applied to populations from the United States, but all aforementioned names remain as valid species in Harbach (5). Because of the lack of COI DNA barcode sequences from correctly identified specimens of *Wyeomyia* in Central America, we have identified Mexican specimens as *Wy. adelpha*/*Wy. guatemala*. This fact highlights yet again the need for expansion of the DNA barcode reference library in combination with revisionary taxonomy.

Although members of the genus *Toxorhynchites* are not of medical importance, their predatory larvae have been employed for biological control with some success (5). We compared the single barcode sequence from *Tx. haemorrhoidalis haemorrhoidalis* (BOLD:ADE6036) obtained in this study with sequences from French Guiana that were identified as this subspecies (BOLD:ACZ4120), as well as to *Tx. haemorrhoidalis superbus* (BOLD:ACZ3966). This comparison revealed a deep split in the NJ tree with average genetic divergence value of 4.35%. Some authors (34) have suggested the presence of several lineages within this species, and the present results support this conclusion.

In contrast to the cases where the DNA barcode results suggested cryptic species, incomplete separation was apparent between *Ae. insolitus* and *Ae. podographicus* (BOLD:ADE8493) and between *Cx. coronator* and *Cx. usquatus* (BOLD:AAN3636). In these cases, interspecific divergence between the species pairs were <1%, so each pair of species was assigned to the same BIN. As expected from their barcode similarity, *Ae. insolitus* and *Ae. podographicus* both belong to the Podographicus complex of *Aedes*, Similarly, *Cx. coronator* and *Cx. usquatus* belong to the Coronator complex of *Culex*. A few other species pairs were assigned to the same BIN, but they can be separated in the NJ tree. For example, *Cx. mollis, Cx. nigripalpus*, and *Cx. pinarocampa* all share a BIN assignment (BOLD:AAF1735), but they form monophyletic clusters in the NJ tree. The close similarity in their sequences suggests that these species are recently diverged or that there has been recent introgression (71). Despite such complexities, the COI barcodes were always useful in narrowing the taxonomic identity of specimens. This was particularly useful in cases where morphological study only allowed a generic assignment, as in *Wyeomyia* sp. (=*Wy*. nr. *complosa*). When resources permit, it is worth supplementing COI DNA barcodes with a nuclear marker such as ITS2 to help clarify cases of uncertainty (32). With the new addition of several mosquito species to its fauna, Chiapas State is now known to host 148 mosquito species, the greatest diversity of any Mexican state, while the Mexican fauna increases to 238 species.

The use of NGS was essential to identify the vertebrate species that served as the source of the blood meals, as a single female can feed on several hosts, creating amplicon pools that cannot be analyzed by Sanger sequencing. Although it is a common practice to employ a separate DNA extraction for blood meal analysis (16, 19), the single RNA extraction performed conformed with protocols established at APHA for the detection of viral pathogens. By omitting DNase treatment, this approach circumvented the need for a separate DNA extraction to allow vector and host identification, saving time, and resources.

A broad range of host species were identified from blood-fed females, including both birds and mammals. *Aedes angustivittatus, Ae. podographicus, Ae. trivittatus, Culex* sp., *Cx. nigripalpus, Ps. albipes*, and *Ps. ferox* each fed on only one or two hosts (Table 5), but collectively fed on a wide diversity of large mammals, birds, and humans. The females of these species are highly anthropophilic, so they can maintain arbovirus circulation in rural or sylvatic settings. For example, the importance of *Ps. albipes* and *Ps. ferox* in the circulation of Venezuelan equine encephalitis virus (VEEV), WNV, and LaCrosse virus in tropical regions has been well-established (62). By contrast to the focused host use of other species, *Cx. quinquefasciatus* fed on a wide range of hosts such as cow, horse, chicken, human, turkey, great-tailed grackle, Virginia opossum, and Muscovy duck, all species common in farmland settings. This result contrasts with other studies; Janssen et al. (44) found humans were its primary host food (63–77%), whereas Estrada-Franco et al. (56) found it fed largely on dogs. Interestingly, (89) found it used diverse hosts in Nevada, USA. Our results suggest that *Cx. quinquefasciatus* is mainly ornithophilic across sylvan communities in Chiapas State, but also feeds on mammals, confirming that it could have an important bridge role in arbovirus transmission (3, 42, 56, 90–93).

There is known circulation of VEEV and St, Louis encephalitis virus in southern Mexico, and WNV antibodies have also been reported in chicken, turkey, and cattle in Chiapas (39, 94–96). Despite these observations, we failed to detect *Flavivirus* or *Alphavirus* RNA using generic primers on both pools of unfed mosquitoes (Table 6) or individual blood-fed specimens (Table 4). It needs emphasis that in regions with high circulation of arboviruses, many thousands of mosquitoes are typically pooled must routinely for effective detection. Viewed from this perspective, the number of samples tested in this study was small involving only 204 pools (Table 4), so we may not have collected a statistically significant number of mosquitoes infected with an arbovirus. As well, loss of the cold chain during the transport of specimens to APHA undoubtedly had a negative effect on any viral RNA that may have been present. As a result, additional collecting should be undertaken in Chiapas to assess viruses that are in circulation.

In conclusion, this study has established that residual DNA in standard RNA extracts can be employed as a template for DNA barcoding to enable vector and host identification. However, we acknowledge that their suggested procedure is still not proven to be effective at detecting RNA based viruses because many samples were not maintained at low temperatures during transport, and we have not tested in detail how DNA in an RNA sample can interfere with the PCR assay in a varied set of samples. In addition, we are aware that usually viral RNA is very low in wild samples originating either from mosquitoes or vertebrates; thus, we advocate for further studies to analyze the effectiveness of this methodology in detecting RNA viruses across a broader range of taxa. Nonetheless, this approach will help to clarify the interactions between insect vectors and both their vertebrate hosts and viral pathogens more efficiently by avoiding the DNA and RNA coextraction from each sample. This, in turn, will provide the essential information needed in order to manage and establish the relevant control strategies against vector borne diseases.

This study has extended understanding of the mosquito fauna in the sylvatic areas of Chiapas State and suggests the presence of cryptic species in nine morphospecies. A broad range of host species was used as a blood meal source by *Cx. quinquefasciatus*, supporting its likely role as a bridge vector for arbovirus transmission. Finally, this study highlights the need to develop a comprehensive DNA barcode molecular library for the mosquito fauna in Mexico and other countries in Central America.

## Data Availability Statement

Detailed specimen records and sequence information (including trace files) were uploaded to the Barcode of Life Database (BOLD—http://www.boldsystems.org) within datasets (projects): DS-MQLC “DNA Barcoding mosquitoes sylvan communities in Mexico (records more than 300 bp) Lacandon Jungle (records <300 bp)”; DS-MQLCJ “DNA Barcoding mosquitoes sylvan communities in Mexico (13- bp shorter sequences).” The Digital Object Identifier (DOI) for the publicly available projects in BOLD is dx.doi.org/10.5883/DS-MQJLC and dx.doi.org/10.5883/DS-MQLCJ. All generated sequences of more than 300 bp have been submitted to GenBank (accession numbers: MT552364—MT552598).200526.

## Ethics Statement

The Animal and Plant Health Agency received permits to carry out surveillance studies on potential infected samples.

## Author Contributions

LH-T, JG-H, AO, SP, PH, AF, and MR-P contribution to the study conception and design. JG-H, AO, EL-S, VG-A, and RM-L material preparation, specimens' collection, and morphological identification of specimens, interpretation for the work. LH-T, SP, PH, NN, EB, and RC-C molecular identification and analysis of sequences. LH-T, AF, PH, and MR-P funding acquisition. LH-T, JG-H, AO, SP, PH, NN, EB, EL-S, VG-A, RM-L, RC-C, AF, and MR-P drafting the manuscript or revising it critically for important intellectual content. All authors contributed to the article and approved the submitted version.

## Conflict of Interest

The authors declare that the research was conducted in the absence of any commercial or financial relationships that could be construed as a potential conflict of interest. The reviewer MH declared a shared affiliation with the authors SP, PH, and NN to the handling editor at time of review.

## Supplementary Material

Supplementary Data Sheet 1 Neighbor-joining tree based on the Kimura two-parameter (K2P) distances showing the arrangement of COI DNA barcodes of 130 bp in length in relation to barcodes of >300 bp for mosquito species collected in sylvan communities in Chiapas State, Mexico. A divergence > 2% may be indicative of separate operational taxonomic units. All barcodes of 130 bp are highlighted in red.

Supplementary Figure 1 Maximum Likelihood tree based on COI DNA barcodes (>300 bp) for mosquito species recorded in sylvan communities in Chiapas State, Mexico. A divergence > 2% may be indicative of separate operational taxonomic units. Values over each node indicate support values. An asterisk (^*^) relates to species from which sequences have been downloaded from BOLD and NCBI databases.

Supplementary Figure 2 Maximum parsimony tree based on COI DNA barcodes (>300 bp) for mosquito species recorded in sylvan communities in Chiapas State, Mexico. A divergence > 2% may be indicative of separate operational taxonomic units. Values over each node indicate support values. An asterisk (^*^) relates to species from which sequences have been downloaded from BOLD and NCBI databases.

Supplementary Table 1 Species, number of sequences, sample ID, process ID, BIN, and country of sequences downloaded from BOLD or NCBI and added to the dataset of COI DNA barcodes (>300 bp) obtained from mosquitoes collected in sylvan communities in Chiapas State, Mexico.

Supplementary Table 2 Percentage of interspecific (between groups) pairwise Kimura two-parameter (K2P) genetic divergence of unique DNA barcodes (658 bp) for 55 species of mosquitoes collected in sylvan communities in Chiapas State, Mexico. Highest pairwise distances (most divergent taxa) and lowest pairwise distances (most closely related taxa) are highlighted in orange and yellow, respectively.
