# Supplementary figures and images for: An Integrated Molecular Approach to Untangling Host–Vector–Pathogen Interactions in Mosquitoes (Diptera: Culicidae) From Sylvan Communities in Mexico
Source: Front Vet Sci. 2021 Mar 10;7:564791. doi: 10.3389/fvets.2020.564791 (PMC7988227; doi:10.3389/fvets.2020.564791)

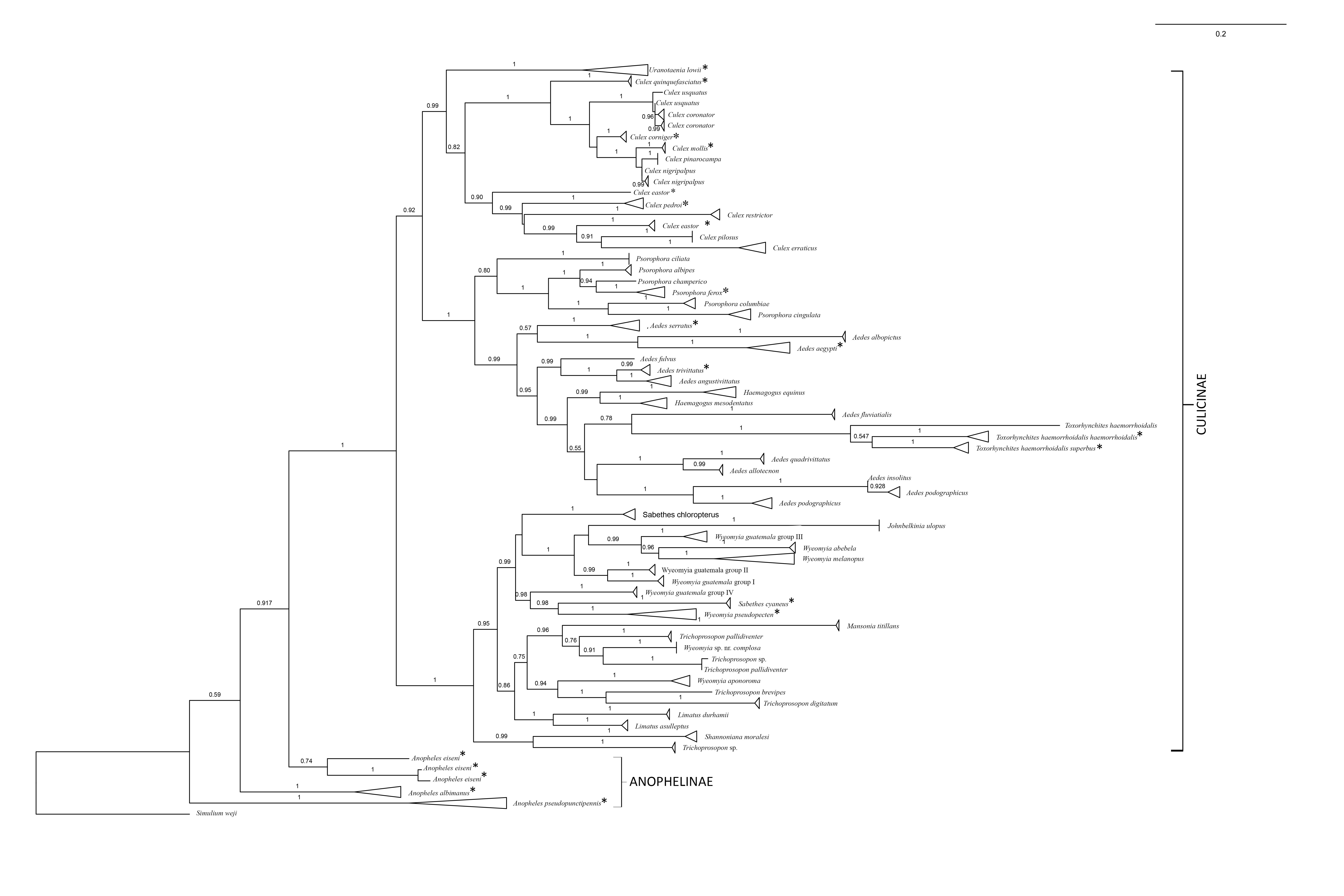

Supplement: Supplementary Figure 1 — Maximum Likelihood tree based on COI DNA barcodes (>300 bp) for mosquito species recorded in sylvan communities in Chiapas State, Mexico. A divergence > 2% may be indicative of separate operational taxonomic units. Values over each node indicate support values. An asterisk (*) relates to species from which sequences have been downloaded from BOLD and NCBI databases. [file Image_1.TIF]

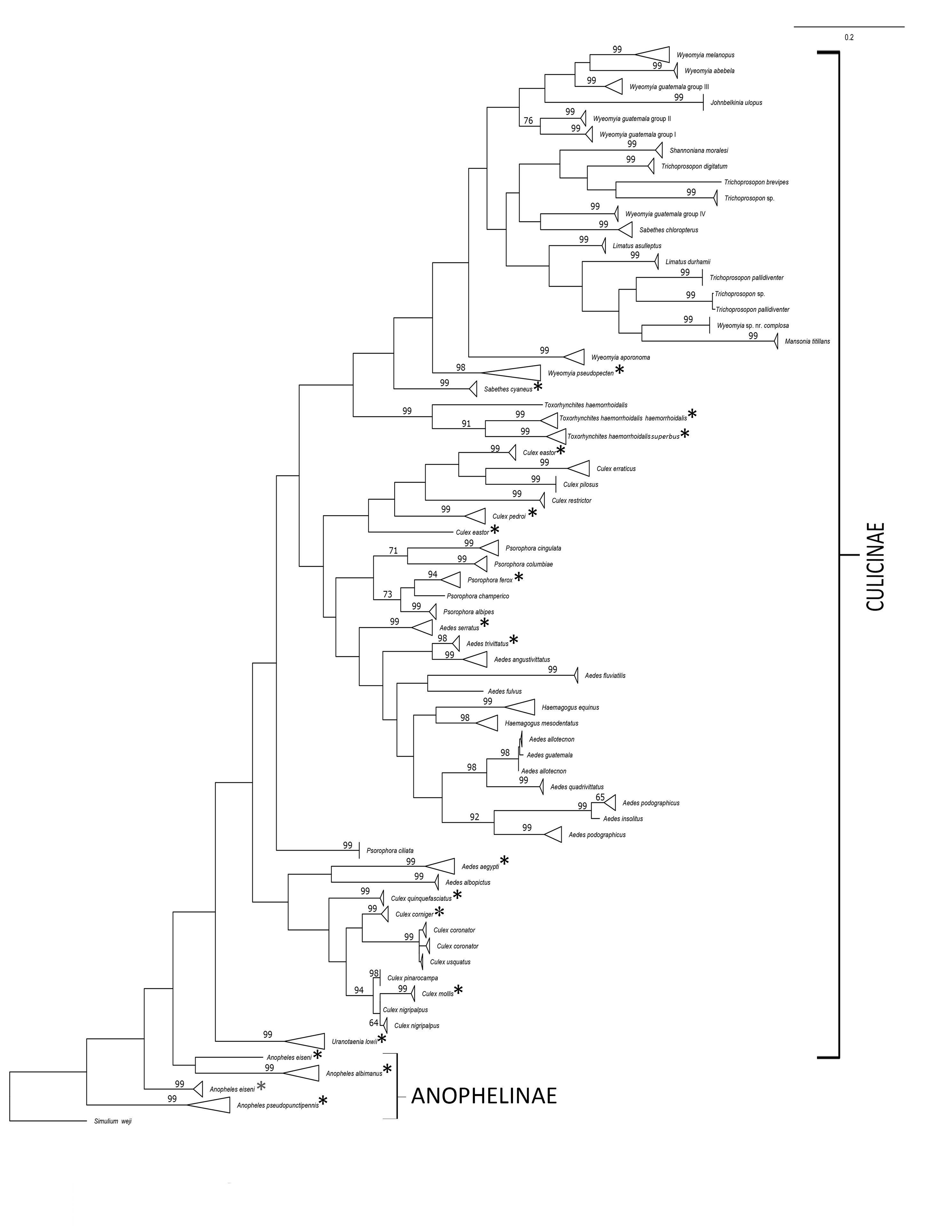

Supplement: Supplementary Figure 2 — Maximum parsimony tree based on COI DNA barcodes (>300 bp) for mosquito species recorded in sylvan communities in Chiapas State, Mexico. A divergence > 2% may be indicative of separate operational taxonomic units. Values over each node indicate support values. An asterisk (*) relates to species from which sequences have been downloaded from BOLD and NCBI databases. [file Image_2.TIF]
