# Supplementary material for: An Integrated Molecular Approach to Untangling Host–Vector–Pathogen Interactions in Mosquitoes (Diptera: Culicidae) From Sylvan Communities in Mexico
Source: Front Vet Sci. 2021 Mar 10;7:564791. doi: 10.3389/fvets.2020.564791 (PMC7988227; doi:10.3389/fvets.2020.564791)

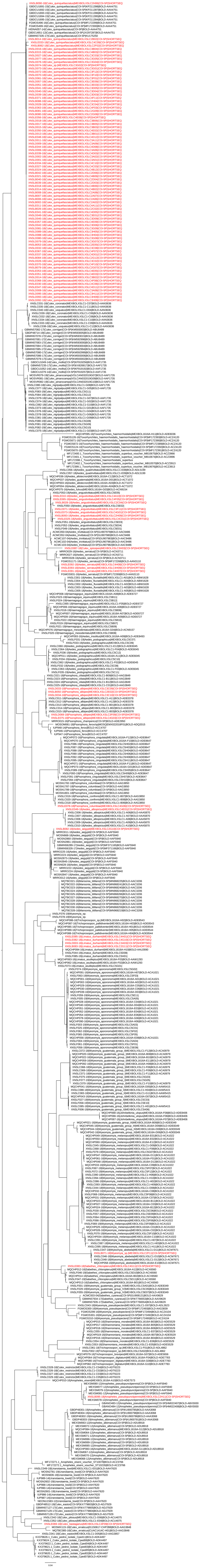

Supplement: Supplementary Data Sheet 1 — Neighbor-joining tree based on the Kimura two-parameter (K2P) distances showing the arrangement of COI DNA barcodes of 130 bp in length in relation to barcodes of >300 bp for mosquito species collected in sylvan communities in Chiapas State, Mexico. A divergence > 2% may be indicative of separate operational taxonomic units. All barcodes of 130 bp are highlighted in red. [file Data_Sheet_1.PDF]
